# Probiotics in Intestinal Mucosal Healing: A New Therapy or an Old Friend?

**DOI:** 10.3390/ph14111181

**Published:** 2021-11-19

**Authors:** Eirini Filidou, George Kolios

**Affiliations:** Laboratory of Pharmacology, Faculty of Medicine, Democritus University of Thrace, 68100 Alexandroupolis, Greece; efilidou@hotmail.com

**Keywords:** probiotics, intestinal wound healing, gut microbiota, mucosal healing, inflammatory bowel disease

## Abstract

Inflammatory bowel disease (IBD), Crohn’s disease, and ulcerative colitis are characterized by chronic and relapsing inflammation, while their pathogenesis remains mostly unelucidated. Gut commensal microbiota seem to be one of the various implicated factors, as several studies have shown a significant decrease in the microbiome diversity of patients with IBD. Although the question of whether microbiota dysbiosis is a causal factor or the result of chronic inflammation remains unanswered, one fact is clear; active inflammation in IBD results in the disruption of the mucus layer structure, barrier function, and also, colonization sites. Recently, many studies on IBD have been focusing on the interplay between mucosal and luminal microbiota, underlining their possible beneficial effect on mucosal healing. Regarding this notion, it has now been shown that specific probiotic strains, when administrated, lead to significantly decreased inflammation, amelioration of colitis, and improved mucosal healing. Probiotics are live microorganisms exerting beneficial effects on the host’s health when administered in adequate quantity. The aim of this review was to present and discuss the current findings on the role of gut microbiota and their metabolites in intestinal wound healing and the effects of probiotics on intestinal mucosal wound closure.

## 1. Introduction

Inflammatory bowel disease (IBD), Crohn’s disease (CD), and ulcerative colitis (UC) are chronic idiopathic inflammatory conditions with a multifactorial background of pathogenesis that remains unclear and requires multiple approaches and complex tools and methods for its elucidation, such as utilized by systems biology [1]. Numerous experimental and genome-wide association studies have revealed many genetic and environmental factors and IBD susceptibility genes that may affect innate immunity, T cell activation, and molecular mediators’ regulation [2,3,4]. Gut commensal microbiota play a crucial role in mucosal immunology mechanisms that are implicated in chronic intestinal inflammation, and several studies have demonstrated a reduction of about 25% of microbiome diversity in patients with IBD and experimental models of colitis [5,6,7,8]. Despite the accumulated evidence on the role of this imbalance, defined as dysbiosis, in IBD, it is not clear whether dysbiosis is a causal factor or the result of chronic inflammation. It is known that active inflammation in IBD disrupts the mucus layer structure and, therefore, its barrier function and colonization sites [9]. On the other hand, it is known that gut microbiota play key roles in the maintenance of homeostasis, and dysbiosis is engaged in the development of chronic inflammation in the intestine [10].

Recently, researchers studying the etiology of IBD have focused on a perverted interplay between mucosal and luminal microbiota [11], highlighting mucosal healing as an emerging therapeutic target. The role of intestinal microbiota in wound healing started to be studied in germ-free mice years ago [12], and recent studies demonstrate that the administration of specific probiotic strains leads to the reduction in inflammation markers and amelioration of colitis in murine colitis models [13]. In this context, recent studies have proven that the attainment of mucosal healing confines serious risks related to IBD [14]. The wound healing process of the gastrointestinal tract is a procedure that rehabilitates homeostasis and involves a complex signaling network of cell migration, proliferation, and epithelial reconstitution [15]. For these reasons, it has been proposed that dysbiosis could be targeted therapeutically with the administration of probiotics. Since the beginning of the last decade, it has been proposed that wound healing could be achieved by using recombinant probiotics [16]. Probiotics are defined as live microorganisms with a beneficial impact on the health of the host when administered in adequate quantity [17], and they have been harnessed as therapeutic approaches in human clinical trials and experimental colitis models [18,19]. Moreover, recent research comes to unchain the mechanisms of mucosal healing through probiotics by identifying specific molecules derived from a probiotic microorganism as a contingent factor for intestinal wound repair in ulcerative colitis [20]. Moreover, Zeissig et al. [21] showed that a vigorous response to biologics, with subsequent mucosal healing, evokes alterations in host transcriptome. Nonetheless, two recent reviews by the Cochrane Gut Group showed that the administration of probiotics did not differ from the placebo in inducing remission in CD and that their possible beneficial effect in maintaining remission in UC is still unclear, as several studies had significant limitations and their results were not strongly supported [22,23], suggesting that more clinical studies are needed in order to verify whether probiotics have an effect in inducing and maintaining remission in IBD. 

During the last years, the manipulation of IBD has been revolved around therapeutic applications that aim to induce and maintain remission, while also confining surgery [24]. Due to the fact that IBD’s etiology is unclear, the drugs that have been developed until now do have not any etiopathologic basis, but they aim to dampen the recusant inflammatory response [25]. These drugs mainly include a wide variety of immunosuppressive agents (e.g., azathioprine), anti-inflammatory compounds (e.g., corticosteroids), and biologics (e.g., anti-TNFα antibody) [26,27,28]. However, current therapy is deficient, as, in many cases, patients relapse, require surgery, and disease recurs after surgery [29]. Recent studies indicate that the restoration of gut microbiota imbalance is important in the management of certain manifestations of chronic intestinal inflammation, and it gives room to probiotics in the quiver of IBD treatments. The aim of this review was to present the current knowledge regarding the role of gut microbiota and their metabolites in intestinal wound healing and to discuss the dynamics of probiotics in the intestinal mucosal wound closure through the restoration of dysbiosis.

## 2. Mucosal Healing in Intestinal Inflammation

Mucosal healing in intestinal inflammation and particularly in IBD, as it is defined by the International Organization of IBD (IOIBD), is the absence of all friability and visible ulcers and erosions in all examined segments of the gut mucosa [30,31]. Mucosal healing, documented via endoscopic scores, combined with clinical remission, has been characterized as deep remission [32]. The term complete remission, which includes histological remission, in addition to mucosal healing and clinical remission, has been suggested as a treatment target in IBD [33]. Histological remission is determined by the absence of polymorphonuclear cells in the crypts and lamina propria, the presence of a normal number of eosinophils, and the absence of plasma cells. However, this definition is more appropriate for UC rather than CD, which is characterized by discontinuous and transmural bowel lesions, and it is still obscure whether mucosal healing also implies complete transmural healing [33]. 

Mucosal healing was traditionally assessed by endoscopic examination. In CD, clinicians usually validate endoscopic mucosal healing by examining four different factors—namely, (a) the location of ulcers on the intestinal surface, (b) the location of other inflammatory lesions on the intestinal surface, (c) the presence or absence of ulcers, and finally, (d) the presence of stenotic areas [34]. In UC, the assessment of endoscopic mucosal healing is more complicated, as several different factors need to be evaluated, including (a) the presence of erythema, friability, ulcers, and erosions; (b) the absence of vascular pattern; (c) the presence of spontaneous bleeding [34].

Recently, deep remission has been defined as clinical remission, followed by mucosal healing and histological remission. In addition, newer markers, such as fecal calprotectin, magnetic resonance enterography, and capsule endoscopy are also used to define clinical remission and mucosal healing [35,36]. Although the definition of mucosal healing in IBD remains controversial, the IOIBD has proposed a definition of histological remission, the absence of neutrophils in crypts and lamina propria, the absence of basal plasma cells, and the reduction in eosinophils in lamina propria to normal counts [33]. Given that CD is a discontinuous and transmural disease, histological remission in patients with CD is less clearly defined compared with patients with UC, in whom histological healing would indicate complete remission and probably be a better clinical outcome, compared with endoscopic and clinical remission [37]. Transmural healing in CD and histological healing in UC should be considered as markers of the extent of remission, while mucosal healing should be treated as an initial event that suppresses the submucosal inflammation, rather than as a sign of complete suppression of gut inflammation.

Mucosal healing seems to improve the clinical course of patients with IBD, by reducing drug therapy, hospitalization, and surgery [38]. However, whether mucosal healing improves the natural history of the disease and the long-term disease-related morbidity still remains unclear. Various studies have shown that deep remission is associated with increased chances of steroid-free clinical remission and decreased risk of disease relapse in patients with CD, while mucosal healing has been shown to reduce the risk of colorectal carcinoma in patients with UC [39,40]. Although there are not many studies, achieving mucosal healing seems to be cost effective, as it results in reduced hospitalization and surgery and improves the quality of life of patients [41], and it should be recognized as the main target for IBD therapy [42].

The intestinal epithelium absorbs nutrients and water from the gut lumen, it is implicated in the fluid and electrolyte homeostasis, and it has to tolerate the commensal microbiota while at the same time combating the pathogenic bacteria [43]. The regular and coordinated performance of these fundamental functions of the intestinal mucosa is based on the fact that it is a highly regenerative tissue, under normal conditions or after damage [44]. The intestinal epithelium normally is rapidly renewed, as epithelial stem cells in the bottom of crypts give rise to absorptive enterocytes and secretory cells, and when the damage is able to be repaired, several adaptive mechanisms occur. These mechanisms include several events, such as the increase in epithelial proliferation, the decrease in apoptosis, the migration of mesenchymal and immune cells in the wounded surface area, and a well-orchestrated inflammatory response that lead to wound healing and prevents a chronic injury and inflammation [45]. 

Following inflammation and injury, the intestinal mucosa undergoes a healing process through complex mechanisms of epithelial restitution, proliferation, and differentiation, and a network of cellular communication of epithelial, mesenchymal, and immune cells, such as macrophages, granulocytes, and lymphocytes [15,46]. This process leads to the recruitment of immune cells and the release of different cytokines that coordinate the trafficking of immune cells with complex interactions with different cellular components via the induction of particular cell signaling pathways [47]. Thus, the wound healing of the intestinal mucosa is a function that participates in the inhibition of the inflammatory response, which results in the damage restoration and contributes to the suppression of inflammation.

The process of intestinal wound healing consists of three successive cellular phases: restitution, proliferation, and differentiation of the epithelium, surrounding the wounded area. However, these steps overlap with each other, and many cellular elements and soluble mediators are involved in more than one phase [48]. The initial response, following intestinal inflammation and mucosal damage, is characterized by a type 1 immune response and the production of pro-inflammatory cytokines. In contrast, the mucosal healing process is a type 2 immune response with increased anti-inflammatory cytokines production that governs tissue regeneration and homeostasis [49]. The first step, termed epithelial restitution, involves epithelial cells migration into the damaged area, forming structures to extend into the denuded mucosa and close the wound. The most important regulator of restitution is transforming growth factor β (TGF-β) that is produced by epithelial cells, myofibroblasts, regulatory T cells, dendritic cells, and macrophages in the gut mucosa [50,51]. Activation of TGF-β enhances restitution by upregulating a number of mediators such as matrix metalloproteinases [52] and vascular endothelial growth factor [53], which promote epithelial cell migration and amino acids such as histidine and arginine that mediate restitution via interaction with Smad signaling [54]. This phase seems to be independent of cell proliferation.

The next phase, proliferation, is mediated by growth factors, including epidermal growth factor, fibroblast growth factor, and keratinocyte growth factor [55,56], and by cytokines such as IL-6, IL-28, IL-33, and IL-22 [57,58,59,60] that increase the number of epithelial cells in order to recover the damaged mucosa and promote immune homeostasis [48]. TLR2 has been found to suppress apoptosis of epithelial cells and promote wound healing by regulating epithelial connexin-43 and trefoil factor 3 expression [61,62]. Another study has shown that interferon-γ induces ligand intercellular adhesion molecule-1 expression in neutrophils and neutrophil binding resulting in increased epithelial cell proliferation, and wound repair [63]. The last phase of differentiation follows the normal process in which intestinal stem cells, located in the crypts, differentiate into secretory to absorptive cell types of progenitors that renew the cellular population of the gut epithelium [64]. This final step of mucosal healing that implicates differentiation and maturation is crucial for the maintenance of the mucosal barrier function [47]. 

### Immune Cells and Soluble Mediators in Mucosal Healing

This sequential process of mucosal healing involves a number of immune and stromal cells of the gut mucosa, communicating and interacting through the secretion of cytokines, growth factors, and conventional gut peptides that are involved in inflamed and restoration processes [42,65]. Neutrophils are the first leukocytes that migrate to sites of mucosal injury, promoting inflammation, in response to the chemokines-rich milieu [66,67]. These cells respond to proinflammatory cytokines by attracting inflammatory monocytes and promoting further inflammation and impairment of the mucosal injury [68]. However, their antimicrobial properties through phagocytosis, production of reactive oxygen species, regulation of the local microenvironment through oxygen metabolism, and the formation of neutrophil extracellular traps (NETs) are essential for wound healing [69,70,71]. Reactive oxygen species generated from neutrophils were found to orchestrate signaling events in epithelial cells contributing to intestinal repair [72]. Depletion of neutrophils or blocking neutrophil invasion in the inflamed gut mucosa, in experimental models of colitis, resulted in aggravation of colitis and impaired restoration of epithelial integrity [73,74].

Despite intestinal mucosa being a large macrophage pool [75], circulating monocytes are rapidly recruited to injured or inflamed areas, increasing the number of tissue macrophages in intestinal inflammation that differentiate into inflammatory M1-like or wound repairing M2-like macrophages [76,77]. Alterations in macrophage differentiation and functionality might contribute to increased susceptibility to IBD [78,79]. Aberrant M1/M2 macrophage polarization and the presence of intestinal Toll-like receptor-responsive macrophages are implicated in the severity and progression of IBD [80,81]. However, due to their heterogeneity, they are implicated in all phases of initiation and restoration of inflammation, including wound repair. Depletion of macrophages in experimental models resulted in increased injury and delayed regeneration and healing, indicating that are necessary for proper epithelial regeneration [82,83]. In addition, macrophages could promote wound repair through the production of cytokines [84], such as IL-10, which possess anti-inflammatory and homeostatic properties [85], and IL-23, an important mediator of wound healing [86]. Another study demonstrated that liver and lymph node sinusoidal endothelial cell C-type lectin (LSECtin)-dependent apoptotic cell clearance by macrophages promotes resolution of inflammation and intestinal regeneration in a model of colitis via the activation of mammalian target of rapamycin (mTOR) [87]. 

Soluble mediators secreted by T lymphocytes play a crucial role in immune and stromal cell communication and cell trafficking during the wound healing process [88,89]. Activated T helper cells (T_H_) produce various cytokines that induce tissue regeneration and healing. T_H_17 and T_H_22 have been shown to produce IL-22 that ameliorates intestinal inflammation [90] and promotes wound healing, via the increase in innate lymphoid cells (ILC3) and mucus production in the intestinal epithelium [91]. Injury of the intestinal mucosa can induce polarization of naïve T cells to T_H_17 cells, via IL-6, TGF-β, and IL-1β signaling, which further expand and produce IL-17 and IL-22, promoting wound healing [92]. Furthermore, γδ T cells are recruited in the site of injury via the expression of CCL20 [93], where they promote healing by producing Keratinocyte growth factor (KGF) in the gut mucosa, which maintains the integrity of intestinal epithelium and is also involved in epithelial cell proliferation and differentiation, which is important in tissue repair [94]. In addition, resident γδ T cells are implicated in wound healing by promoting proliferation and migration of stem cells in the side of injury [95]. Another study has shown that mice lacking γδ T cells had a reduced ability to repair the gut injury in a model of Dextran Sulfate Sodium (DSS)-induced colitis [96]. However, T lymphocytes mediators’ production during inflammation and tissue injury could also worsen the inflammatory process, if not tightly controlled.

Treg cells with a stable expression of Foxp3 were initially considered as the main regulatory T cell population. Over the last years, a heterogeneity of different Treg cell populations has been reported. Suppressor T cells could inhibit the effect of T_H_ cells via the production of anti-inflammatory and immunomodulatory cytokines [97,98]. Experimental studies from various organs in mice have shown that the depletion of Tregs deteriorates the clinical outcome by increasing the inflammation and inhibiting wound healing [99,100]. There is evidence that Treg-produced fibroblast growth factor (FGF) and IL-17 decrease the accumulation of pro-inflammatory macrophages and are also implicated in gene regulation of intestinal epithelium’s repairment [101,102]. Another study has shown that Foxp3^+^Tregs might promote mucosal healing in intestinal inflammation and injury via vascular endothelial growth factor receptor 1 tyrosine kinase (VEGFR1-TK) signaling, as mucosal repair in DSS-induced colitis is impaired in VEGFR1-TK knock-out mice [103]. On the other hand, expansion of regulatory T cells has been reported to maintain mucosal healing in UC [104]. Data from experimental studies have shown that commensal microbiota regulate the generation of regulatory T cells from microbial activated effector T cells [105]. Accumulation of Tregs in the lamina propria of the large and small intestine has been found to be affected by changes in gut microbiota, as it was found impaired in germ-free or antibiotic-treated mice, and fecal transplantation from normal mice increased the number of Tregs [106], while probiotic administration has been found to modulate the functional metabolism of regulatory T cells via the regulation of dysbiosis [107]. 

Innate lymphoid cells (ILCs), another important cell population of the intestinal mucosa [108], apart from their contribution to IBD, promote resolution of intestinal inflammation and mucosal healing [109]. The ILC3s subset is the main source of IL-22 after induction by IL-23 during intestinal damage, which protects intestinal stem cells from immune-mediated responses and activates them to promote would repair [110,111]. In addition, the ILCregs subset promotes wound healing via the secretion of IL-10, suppressing activated ILC1s and ILC3s subsets [112]. Recently, another study demonstrated that GPR34 receptor deficiency in the ILC3s subset decreased IL-22 production and tissue repair in colon and skin injury in mice. Expression of GPR34 receptor in ILC3s triggers intestinal mucosa healing, upon recognition of dying neutrophils [113]. 

The mucosal healing process in intestinal inflammation repairs mucosal integrity and maintains the epithelial barrier with important clinical benefits. Although certain mechanisms and immune cells implicated in the process of wound repair are well studied, the overall picture of the interplay between cellular components and mediators has not been clarified.

## 3. Gut Microbiota as Mediators of Mucosal Healing

The contribution of gut microbiota to the wound healing process of the intestinal lumen is highlighted by several in vitro and in vivo studies [114,115,116,117,118]. Pull et al. [83] showed that intestinal wound healing in germ-free mice was greatly affected due to a significant decrease in the proliferation rate of colonic epithelial stem cell progenitors. In another study, it was reported that during regeneration of epithelial wounds, a consortium of anaerobic and mucinophilic bacteria transiently reside and repopulate in the close proximity of the murine intestinal epithelial cells, suggesting that they might be actively implicated in the regeneration process [119]. 

However, mechanistically, it is not well understood how the intestinal resident microbiota influences the efficient maintenance and/or repair of the epithelial barrier [46], and it seems that this repair process is orchestrated by a coordinated network of different cellular, immunological, biochemical, and also microbial influences [120]. One study suggests that commensal bacteria regulate cell migration and restoration of intestinal barrier functions via induced generation of ROS in epithelial cells [121], while another study by Rakoff- Nahoum et al. [122,123] demonstrated that commensal bacterial recognition by Toll-like receptors (TLRs) plays a crucial role in the protection of intestinal epithelia. Another possible mechanism by which gut microbiota favor wound healing is described in the study by Alam et al. [117,119]. They showed that specific types of bacteria interact with N-formyl peptide receptors on intestinal epithelial cells, resulting in the production of reactive oxygen species that ultimately activate kinases associated with enterocyte proliferation and migration. 

In a more recent study, the gut microbiota have been linked to the favorable immunological responses that occur during wound healing after DSS-induced damage. In particular, when IL-36γ, an interleukin of the IL-1 family known to positively contribute to wound healing [124], was undetectable in germ-free mice, or its receptor was genetically deleted, they presented with significant wound healing impairment following DSS [125]. Along the same lines, Song et al. [101] showed that during DSS treatment, the dysregulated microbiota induce the expression of TGFβ1, activating Treg cells to secrete FGF2, which, along with IL-17, leads to a gene signaling pathway to repair the damaged epithelium. 

Another possible way that microbiota exert their therapeutic properties on wound healing is via their exopolysaccharides. Zhou et al. [126] showed that the exopolysaccharides from *Lactobacillus plantarum* could positively influence wound healing as they promoted the goblet cell differentiation, through the induction of the expression of c-Jun/Muc2 signaling pathway. Apart from the immunological studies, Abo et al. [127] demonstrated that early life microbiota influence intestinal development, through the activation of erythroid differentiation regulator-1 that promotes intestinal stem cell proliferation and regeneration. All these aforementioned studies seem to conclude to the same principle, i.e., microbiota have active roles in wound healing either directly through their interactions with the host’s cells or indirectly, through various signaling pathways.

As already mentioned, gut microbiota influence tissue repair through direct interactions with the host’s cells, but also through the secretion of microbial metabolites (Table 1). 

Alexeev et al. [128,129] reported that microbial-derived indoles assist during epithelial damage and promote tissue healing via the induction of IL-10 signaling and the inhibition of the excessive neutrophil myeloperoxidase production. In the same vein, Sung et al. [130] identified another microbial metabolite, the tuberonic acid, that exerts anti-inflammatory properties and has a beneficial role during epithelial tissue repair. In addition, it has been noted that an imbalanced gut microbiota composition, which, in turn, results in altered microbial metabolite production, contributes to mucus dysfunction, suggesting that specific microbes favor the development and function of the mucus layer [11]. It has been found that germ-free mice develop a thin layer of mucus, which can expand in volume and resemble that of the wild-type mice when commensal bacterial colonization is established [136]. More specifically, Wrzosek et al. [131] reported that when *Bacteroides thetaiotaomicron* and *Faecalibacterium prausnitzii*, two short-chain fatty acids (SCFA)-producing bacteria, were introduced to germ-free mice and colonized their guts, it resulted in an increase in goblet cell differentiation and mucus production. Therefore, microbial metabolites seem to have various effects, influencing the architecture and functions of the whole intestinal barrier. SCFA’s positive effect on wound healing has also been highlighted by Park et al. [132] and Kelly et al. [133], as they showed that these metabolites enhance epithelial proliferation and differentiation, and support the epithelial barrier upon tissue damage. 

Another very common microbial metabolite, lactate, has been found to exhibit healing properties on the intestinal mucosa both in vitro and in vivo. Specifically, L-lactate treatment was found to promote the migration rate of murine intestinal epithelial cells by enhancing their mitochondrial ATP production and to ameliorate colitis in mice, by inducing the expression of Cdc42 and Pak1, two factors associated with intestinal epithelial cell migration [134]. These results suggest that the communication between the host and the microbiota does not depend just on cellular interactions, but reach deeper, as microbiota’s secreted factors actively influence the host’s cellular processes.

The beneficial properties of microbial metabolites in wound healing are further supported by the study of Scott Lee et al. [135]. In this study, the authors investigated the effects of microbial-derived purines in DSS-colitis and found that purines could protect against colitis, by altering the metabolic profile of the treated mice and by enhancing ATP biosynthesis. On the cellular level, these findings were translated to enhanced epithelial proliferation and barrier integrity and reduced apoptosis and thus improved intestinal wound healing, suggesting that microbiota directly contributes to the host’s energy sources [20]. 

Apart from the direct effects, microbiota’s metabolites can also indirectly contribute to the process of wound healing. The long-chain polyphosphate from *Lactobacillus brevis* has been found to enhance the epithelial barrier’s integrity and to promote epithelial regeneration through platelet accumulation at the sites of the wound. Specifically, the supernatant of platelets, which have been previously exposed to the long-chain polyphosphate, could significantly enhance wound healing and epithelial regeneration through the induction of mitogen-activated protein kinase in the epithelial cells [137].

## 4. Therapeutic Applications with Specific Probiotic Strains for the Accomplishment of Mucosal Healing

**Figure 1:**
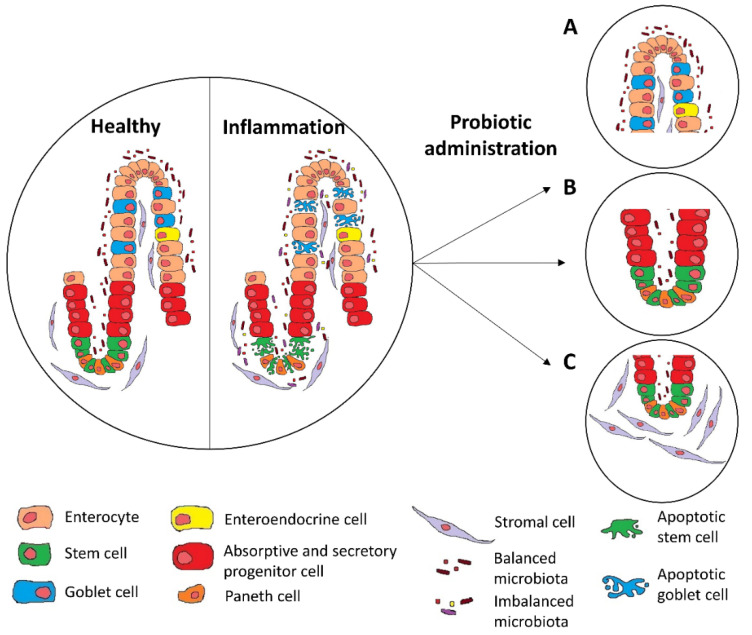
Probiotic administration under inflammatory conditions results in increased goblet cells replenishment (**A**), epithelial crypt regeneration (**B**), and fibroblast migration (**C**).

Since several studies have shown that microbiota have positive effects on mucosal healing; applications with probiotics have also demonstrated promising results [138], with most of the findings regarding their effect on epithelial regeneration and fibroblast migration (Figure 1). 

In particular, experiments on mice using the probiotic strain *Lactobacillus Rhamnosus* CNCM I-3690 showed that goblet cells were replenished and mucus production was amplified [139]. In another study, Toumi et al. [140] administrated for a week Ultrabiotique (*Lactobacillus acidophilus, Bifidobacterium lactis, L. plantarum,* and *Bifidobacterium breve*) to mice, and showed an augmentation of mucus production and goblet cells per crypt, suggesting that probiotics may be a promising therapeutic intervention in situations that require immediate mucosal healing. 

The positive effects of probiotics on wound healing are also highlighted by a recent study using a probiotic mixture containing *L. acidophilus, L. plantarum, L. rhamnosus*, and *Enterococcus faecium*. In this study, the colonic media of patients with UC that previously received the probiotic mixture significantly increased the wound healing rate of epithelial cells in vitro and improved the integrity of their tight junctions’ formation, possibly through the increased production of butyrate [141]. *L. rhamnosus* or *L. plantarum* alone has also been shown to promote mucosal wound healing [117,142], suggesting that even when administrated alone, these probiotics have indeed key roles in tissue repair. *L. rhamnosus GG* has also been shown to restore the epithelial integrity after alcohol treatments through the induction of hypoxia-inducible factor (HIF) in the epithelial cells [143], suggesting that probiotics might also have a protective effect on alcoholic liver disease (ALD). 

In addition, p40, a soluble protein secreted by *L. rhamnosus GG*, has been shown to be directly implicated in the mechanisms by which this strain protects epithelial integrity. Specifically, p40 induces mucin production in the epithelial cells and also prevents epithelial damage and apoptosis caused by pro-inflammatory cytokines [144,145,146,147]. Aside from the well-known effects of *Lactobacillus*, a recent study has highlighted the promising beneficial effects of *Christensenella minuta*. Kropp et al. [148] showed that the strain DSM 22607 of *C. minuta* could ameliorate inflammation and restore the epithelial integrity in vitro, and these results were further verified in vivo. Using two different colitis models, administration of *C. minuta* DSM 22607 resulted in the restoration of colonic epithelial architecture, with decreased signs of inflammation and immune cell infiltration, suggesting that numerous different microbes exert beneficial effects, and more studies are in need. In another study, van der Lelie et al. [149] manufactured two different probiotic mixes consisting of either 17 or 11 different strains and investigated their effect on preventing and reversing experimental colitis. They found that both blends had the ability to reverse colitis and microbial dysbiosis and ultimately, enhance wound healing and restore the intestinal architecture. Therefore, one could argue that a richer probiotic blend could better promote wound healing as it would more accurately resemble the healthy microbiota composition. 

Apart from the positive effects of probiotics on epithelial cells, Im et al. [150] showed that the condition media of the probiotic *Bacillus polyfermenticus* could also beneficially influence endothelial cells by promoting cell migration, permeability, and tube formation. Along the same lines, Dharmani et al. [151] reported that a mix of eight different probiotic bacteria promoted ulcer healing in rats, through the induction of VEGF. These findings suggest that probiotics could induce epithelial regeneration, in addition to promoting endothelial proliferation and angiogenesis. 

In another interesting study employing a mouse model of intestinal ulcers, Yu et al. [152] administrated a probiotic strain of *Escherichia coli* fused with epidermal growth factor (EGF) in mice and observed increased wound healing of the epithelial layers and decreased disease severity when compared with controls, suggesting that the beneficial effects of probiotics could be further enhanced by the engineered addition of various growth factors. In another study, Praveschotinunt et al. [153] engineered an *E. coli* strain that was able to secrete curli nanofibers and to significantly enhance wound healing both in vitro and in vivo, with no signs of pathogenesis, showing again that the combination of bioengineering and microbial biotechnology could greatly favor the positive results seen by the probiotics applications. Regarding the notion of enhancing the beneficial effects of probiotics, Costanzo et al. [154] in vitro treated epithelial cells with a mixture of Krill oil, Vitamin D and *Lactobacillus reuteri* and observed improved wound healing and cell–cell adhesion during inflammatory conditions, suggesting that a more complete diet could greatly ameliorate the consequences of intestinal inflammation.

Apart from the probiotics, the use of prebiotics, substances that positively influence the microbiota’s growth, has also been found to enhance wound healing. In particular, inulin and galactooligosaccharides (GOS), two oligosaccharides, can promote SCFA production by the microbiota and, ultimately, support the host’s epithelial wound healing [155,156]. Hajjar et al. [157] showed that both inulin and GOS diet supplementation resulted in improved wound healing in mice that underwent surgical colonic anastomosis, with increased signs of re-epithelialization and collagen deposition and lower activities of metalloproteinases. 

Although probiotics, in general, have a positive impact on gut immunology and wound healing, one should not forget that probiotics are microorganisms usually administrated as blends consisting of different strains and, therefore, have different effects on the host depending on the type of mixture or the administrative dose. Otte et al. [158] showed that different strains of probiotic mixtures have various effects on the epithelial expression of cyclooxygenase-2 (COX) 2, an important inflammatory mediator, which is also implicated in wound healing. In addition, *Debaryomyces hansenii*, a yeast species, has been found elevated in inflamed mucosa tissues of CD patients, and further research revealed that it plays an active role in obstructing wound healing, suggesting again not all microbiota acts in a positive way for the host and that balance is the key [159]. Therefore, it is only obvious that more research is needed in order to elucidate the functions and mechanisms of each strain alone and in the presence of other ones.

## 5. Conclusions

In conclusion, although it is still not clear whether the observed microbiota dysbiosis comprises the cause or the result of IBD, it is well-known that the chronic inflammation and the subsequent epithelial barrier damage lead to dysregulated interactions between the host and the microbiota [46]. Nonetheless, microbiota can also have beneficial effects on the host, such as reduced inflammation and increased epithelial architecture restoration and wound healing (Table 2). 

Given the fact that a proportion of patients with IBD either do not respond or develop adverse effects to current modern biological treatments, alternative therapeutic strategies employing probiotics, prebiotics, and synbiotics are more and more investigated [160]. Probiotics that are naturally found in various dairy products, such as cheese and yogurt, have been a part of our diet for centuries and have long played a positive role in mucosal gut homeostasis. It is, therefore, crucial that more studies should be conducted in order to elucidate the interactions between the host and microbiota that result in increased mucosal healing, as well as the mechanisms by which the probiotics exert their beneficial effects. Although much remains unclear, one certain fact is that probiotics should not be considered as simple diet supplements but as potential therapeutic factors that, when administrated correctly, can lead even to amelioration of colitis and increased mucosal wound healing.

## Figures and Tables

**Table 1:** Microbiota’s metabolites and their effect of on intestinal wound healing.

Metabolite	Effect	Reference
Indoles	Induction of IL-10 signaling and inhibition of excessive neutrophil myeloperoxidase production	[128,129]
Tuberonic acid	Prevents LPS-induced inflammation by decreasing the levels of the proinflammatory cytokines TNF-α, IL-6, and IL-1β and by increasing the anti-inflammatory cytokine, IL-22	[130]
Short-chain fatty acids (SCFA)	Increase goblet cell differentiation, mucus production, enhance epithelial proliferation and differentiation, and support the epithelial barrier upon tissue damage	[131,132,133]
L-lactate	Promotes migration of intestinal epithelial cells, by enhancing their mitochondrial ATP production. Ameliorates colitis in mice, by inducing the expression of Cdc42 and Pak1.	[134]
Purines	Protect against colitis, by altering the metabolic profile and enhancing the ATP biosynthesis. Enhance epithelial proliferation and barrier integrity.	[20,135]

**Table 2:** The effects of probiotics on intestinal wound healing.

Probiotics	In Vivo or In Vitro Model	Outcome	Reference
*L. Rhamnosus CNCM I-3690*	DNBS-colitis mouse model	Goblet cell replenishment and mucus production amplification	[139]
*L. acidophilus, B. lactis, L. plantarum* and *B. breve*	DSS-colitis mouse model	Goblet cell replenishment and mucus production amplification	[140]
*L. acidophilus, L. plantarum, L. rhamnosus* and *E. faecium*	Caco-2 epithelial cell line	Increased wound healing rate and improved tight junction formation	[141]
*L. rhamnosus GG*	DSS-colitis in transgenic mice, and SK-CO15 epithelial cell line	Increased wound healing	[117]
*L. plantarum*	Gastric ulcers model through luminal application of acetic acid	Increased fibroblast migration and proliferation	[142]
*L. rhamnosus GG*	Alcoholic liver mouse model and Caco-2 epithelial cell line	Restoration of epithelial integrity through the induction of HIF in the epithelial cells	[143]
*B. polyfermenticus*	DSS-colitis mouse model and human intestinal microvascular endothelial cells	Increased wound healing and enhanced endothelial cell migration, permeability, and tube formation	[150]
*L. acidophilus, L. plantarum, L. casei,* and *L. delbrueckii, B. breve, longum,* and *infantis,* and *Streptococcus salivarius*	Gastric ulcers model through luminal application of acetic acid	Promotion of ulcer healing through the induction of VEGF	[151]
*E. coli fused* with EGF	DSS-colitis mouse model	Increased wound healing of the epithelial layers and decreased disease severity	[152]
*L. reuteri*	Caco-2 and HT29 epithelial cell lines	Improved wound healing and cell–cell adhesions	[154]

## Data Availability

No new data were created or analyzed in this study. Data sharing is not applicable to this article.
